# A new species of *Voria* Robineau-Desvoidy (Diptera: Tachinidae) from Area de Conservación Guanacaste in northwestern Costa Rica

**DOI:** 10.3897/BDJ.5.e20123

**Published:** 2017-12-05

**Authors:** AJ Fleming, D. Monty Wood, M. Alex Smith, Tanya Dapkey, Winnie Hallwachs, Daniel Janzen

**Affiliations:** 1 Agriculture and Agri-Food Canada, Ottawa, Canada; 2 University of Guelph, Guelph, Canada; 3 Department of Biology, University of Pennsylvania, Philadelphia, United States of America

**Keywords:** caterpillar, tropical, Voriini, Noctuidae, parasitoid, fly, rainforest, dry forest, cloud forest, ACG

## Abstract

**Background:**

We describe a new species in the genus *Voria* Robineau-Desvoidy, 1830 (Diptera: Tachinidae: Voriini) from Area de Conservación Guanacaste (ACG) in northwestern Costa Rica. It was reared as part of an ongoing inventory of wild-caught caterpillars spanning a variety of moth and butterfly families (Lepidoptera). Our study provides a concise description of the new species using morphology, life history, molecular data, and photographic documentation. In addition to the new species, we provide a diagnosis of the genus as well as new data relating to host use.

**New information:**

The following new species of *Voria* is described: *Voria* *erasmocoronadoi* Fleming & Wood **sp. n.**

The following are proposed by Fleming & Wood as new synonyms of *Voria*: *Xenoplagia* Townsend, 1914 **syn. n.**, *Hystricovoria* Townsend, 1928 **syn. n.**, *Afrovoria* Curran, 1938 **syn. n.**, and *Anavoria* Mesnil, 1953 **syn. n.**, and *Itavoria* Townsend, 1931 **syn. n.**

The following new combinations are proposed as a result of the new synonymies: *Voria bakeri* (Townsend, 1928), **comb. n.** and *Voria setosa* (Townsend, 1914), **comb. n.**The authors also propose *Voria pollyclari* (Rocha-e-Silva, Lopes & Della Lucia, 1999), **comb. n.** based on the morphology of the holotype.

## Introduction

Generally speaking, the tribe Voriini (Diptera: Tachinidae: Dexiinae) can be characterized by two main traits, namely: the obliquely angled sinusoidal hind crossvein, and the long ribbon-like phallus (Thompson 1961). While these characters prove useful to separate most of the voriines from other tribes, this minimalist approach is not a perfect fit, with some genera in the tribe having one but not both of these traits. A more general concept of the Voriini can be gleaned from the works of Cortés and González (1989) and Thompson (1961), both of which characterize the tribe as follows: conical head profile (wider at level of of base of pedicel than at level of vibrissa); frons broad in both sexes; no black setae beyond occipital row; both males and females with proclinate orbital setae; prosternum bare; ocellar setae well-developed, divergent and proclinate; facial ridge bare; anepimeral seta absent, or poorly developed so as to appear hair-like; infrasquamal setae present; apical scutellars strong and decussate; DM-CU crossvein very oblique; R_4+5_ setulose from fork up to or beyond crossvein R-M; stub of vein M extending past the bend of vein M; middorsal depression of syntergite 1+2 (ST1+2) reaching posterior margin; phallus elongate and frequently ribbon-like (Thompson 1961). While there has been much work done on the tribe Voriini (Cortés and González 1989), no major taxonomic work focusing on *Voria *Robineau-Desvoidy, 1830 has yet been published, and the genus remains poorly understood. A major impediment to revising this genus is that the species are all extremely similar morphologically, with only subtle differences in the coloration of the fronto-orbital plate and some minor differences in the terminalia. However, despite their morphological similarities, when examined at a molecular level, they show a great deal of variation among geographically isolated populations, suggesting the existence of a complex of morphologically very similar, yet distinct, species. 

The genus *Voria* was originally erected for *Voria* *latifrons* Robineau-Desvoidy, 1830, which was described based on an unspecified number of specimens (males and females) collected on flowers of *Heraclæum spondylium* L. (Apiaceae) found growing in a prairie in Gentilly (a suburb of Paris, France). Townsend (1939) synonymized *Vorialatifrons*with *Tachina ruralis* Fallén, 1810 , while also placing the genus *Plagia *Meigen, 1838, in synonymy with *Voria*. To date, five names have been sunk into synonymy with *Voria ruralis*, increasing its range from Palearctic only to cosmopolitan. Evidence from DNA barcodes suggests the existence of a great deal of molecular variation among the different populations of this species. As a result, we can only reasonably describe *V. ruralis* as a species complex. DNA barcoding of the holotype is required for a proper definition of *V. ruralis*, and a more in-depth study is needed to differentiate the individual species. The elucidation of the *V. ruralis* species complex falls outside the scope of this paper, where we will only be providing information regarding a single new species described from Area de Conservación Guanacaste (ACG), northwestern Costa Rica (http://www.acguanacaste.ac.cr). Like many other genera of Voriini, species of *Voria* parasitize caterpillars of Lepidoptera, specifically in the families Noctuidae and Uraniidae (Arnaud 1978).

The new species of *Voria *described herein is based on specimens collected during the ongoing inventory of the tri-trophic relationships between herbivores, host plants and parasitoids within the dry, rain, and cloud forests of the terrestrial portion of ACG (Janzen and Hallwachs 2011, Fernandez-Triana et al. 2014, Janzen and Hallwachs 2016). Here, we build on the existing knowledge and base the new species on differences in external morphology, COI (coxI or cytochrome *c* oxidase I) gene sequences, and male terminalia, and discuss its host species. As the inventory is continually growing, the information provided in this paper should not be taken as conclusive on the total number of species of *Voria *present in ACG or Costa Rica, from which only *V. ruralis* had been recorded prior to our study.

This paper is part of a larger effort to describe new species reared during the ACG inventory (Fleming et al. 2014a, Fleming et al. 2014b, Fleming et al. 2015a, Fleming et al. 2015c, Fleming et al. 2015b, Fleming et al. 2015d, Fleming et al. 2016a, Fleming et al. 2016b, Fleming et al. 2017). This series of taxonomic papers will represent the foundation for subsequent, detailed ecological and behavioral studies extending across ACG ecological groups, whole ecosystems, and taxonomic assemblages much larger than that represented by a genus.

## Materials and methods

### Project aims and rearing intensity

All reared tachinid specimens were obtained from host caterpillars collected in ACG (Smith et al. 2006, Smith et al. 2007, Smith et al. 2008, Janzen et al. 2009, Smith et al. 2009, Janzen and Hallwachs 2011, Smith et al. 2012, Janzen and Hallwachs 2016). ACG's 125,000+ terrestrial hectares include portions of the provinces of Alajuela and Guanacaste, along the dry forested northwestern coast of Costa Rica and inland to the Caribbean lowland rainforest. ACG comprises three different ecosystems and their intergrades, ranging from sea level up to 2,000 m. The tachinid rearing methods are described at http://janzen.bio.upenn.edu/caterpillars/methodology/how/parasitoid_husbandry.htm. Since its inception, this inventory has reared over 750,000 wild-caught ACG caterpillars. Any frequencies of parasitism reported here need to be considered against this background inventory. Comparative details of the parasitism ecology of these flies will be treated separately in later papers, once the overall alpha-taxonomy of ACG caterpillar-attacking tachinids is more complete.

### Descriptions and imaging

The description of the new species is complemented with a series of color photos, to illustrate some of the morphological characters described. The morphological terminology used follows Cumming and Wood (2009). All dissections and photography were carried out following the methods detailed in Fleming et al. (2014a). Landmark body structures, measurements and examples of parts of the terminalia are illustrated in Fig. 1.

### Voucher specimen management

All caterpillars reared from the ACG inventory receive a unique voucher code in the format yy–SRNP–xxxxx. Any parasitoid emerging from a caterpillar receives the same voucher code as a record of the rearing event. If and when the parasitoid is later dealt with individually it receives a second voucher code unique to it, in the format DHJPARxxxxxxx. These voucher codes assigned to both the host and its parasitoids may be used to obtain the individual rearing record at http://janzen.bio.upenn.edu/caterpillars/database.lasso.

To date, all DHJPARxxxxxx-coded tachinids have had one leg removed for DNA barcoding at the Center for Biodiversity Genomics, University of Guelph, Ontario, Canada. All successful barcodes and collateral data are first deposited in the Barcode of Life Data System (BOLD, www.boldsystems.org) (Ratnasingham and Hebert 2007), and later migrated to GenBank. Each barcoded specimen is also assigned unique accession codes from the Barcode of Life Data System (BOLD) and GenBank, respectively.

Inventoried Tachinidae were collected under Costa Rican government research permits issued to DHJ and WH, and exported from Costa Rica to Philadelphia, en route to their final depository in the Canadian National Insect Collection in Ottawa, Canada (CNC). Tachinid identifications for the inventory were done by DHJ and WH in coordination with a) visual inspection by AJF and DMW, b) DNA barcode sequence examination by MAS and DHJ, and c) correlation with host caterpillar identifications by DHJ and WH through the inventory itself. The date of collection is the date of eclosion of the fly, not the date of capture of the caterpillar. This is because the fly eclosion date is much more representative of the time when that fly species is on the wing than is the time of capture of the host caterpillar, and therefore a date that is appropriate for comparison with adult-caught specimens in other museum collections. The collector listed on the label is the parataxonomist who found the caterpillar, rather than the person who retrieved the newly eclosed fly from its rearing container. Holotypes of all tachinid parasitoids collected by the ACG inventory, including that of the species newly described herein, are deposited at CNC.

### Acronyms for depositories

CNC – Canadian National Collection of Insects, Arachnids and Nematodes, Ottawa, Canada

ETHZ – Erdgenössische Technische Hochschule-Zentrum, Zurich, Switzerland

IZCAS – Chinese Academy of Sciences, Institute of Zoology, Beijing, China

MLPA – Museo de la Plata, Universidad Nacional de La Plata, La Plata, Argentina

MNHL – Museum d'Histoire Naturelle de la Ville de Lille, Lille, France

MRSN – Museo Regionale di Scienze Naturali di Torino [collection formerly housed at Museo di Zoologia, Istituto di Zoologia e Anatomia Comparata, Università di Torino – MZUT], Turin, Italy

MZLU – Museum of Zoology, Lund University, Lund, Sweden

NHMUK – Natural History Museum, London, United Kingdom [formerly British Museum (Natural History)]

NHRS – Naturhistoriska riksmuseet, Stockholm, Sweden

SANC – South African National Collection of Insects, Pretoria, South Africa

SDEI – Senckenberg Deustsches Entomologisches Institut, Müncheberg, Germany

SEMK – Snow Entomological Museum, University of Kansas, Lawrence, Kansas, USA

UFVB – Museu Regional de Entomologia, Universidade Federal de Viçosa, Viçosa, Minas Gerais, Brazil

USNM – National Museum of Natural History, Washington, D.C., USA (formerly the United States National Museum)

### Interim names of undescribed host species

Names of undescribed host species follow a standardized, interim naming system used for taxonomic units considered as distinct species not yet formally identified or scientifically described but reliably identified by DNA barcodes. The interim names are given in the format "*Eois* Janzen52" or "*Caviria regina*DHJ01", where the "species epithet" is either composed of the name of the taxonomist who identified the species and a number or the name of a species-group followed by a code. This prevents confusion with already described species while maintaining traceability of each undescribed species within the ACG project.

### DNA Barcoding

The standard DNA barcode region for animals (5’ cytochrome c oxidase I (CO1) gene) (Hebert et al. 2003) was examined from all four specimens of ACG *Voria*. DNA extracts were obtained using a standard glass fiber protocol (Ivanova et al. 2006) from single legs. Using standard barcode region primers for insects (LepF1–LepR1) and following established protocols for production and quality control (Smith et al. 2006, Smith et al. 2007, Smith et al. 2008, Smith et al. 2009), we amplified 658 bp near the 5’ terminus of the CO1 gene. The Barcode of Life Data System (BOLD) (Ratnasingham and Hebert 2007) can be consulted for information (including GenBank accession codes) associated with each sequence, by using the persistent DOI dx.doi.org/10.5883/DS-ASVORIA. 

## Taxon treatments

### Voria

Robineau-Desvoidy, 1830

Voria Robineau-Desvoidy, 1830: 195. Type species: *Voria latifrons* Robineau-Desvoidy, 1830 [=*Tachina ruralis* Fallén, 1810], by monotypy.
Plagia Meigen, 1838: 201. Type species: *Tachina verticalis* Meigen, 1824 [=*Tachina ruralis* Fallén, 1810], by subsequent designation of Rondani (1856).
Xenoplagia Townsend, 1914: 13. Type species: *Xenoplagia setosa* Townsend, 1914, by original designation. **Syn. n.**
Hystricovoria Townsend, 1928: 395. Type species: *Hystricovoria bakeri* Townsend, 1928, by original designation. **Syn. n.**
Itavoria Townsend, 1931: 475. Type species: *Itavoria aurescens* Townsend, 1931, by original designation. **Syn. n.**
Afrovoria Curran, 1938: 5. Type species: *Afrovoria munroi* Curran, 1938 [=*Hystricovoria bakeri* Townsend, 1928], by original designation. **Syn. n.**
Anavoria Mesnil, 1953: 170 (as subgenus of *Voria* Robineau-Desvoidy, 1830). Type species: *Voria *(*Anavoria*) *indica* Mesnil, 1953 [=*Hystricovoria bakeri* Townsend, 1928], by monotypy. **Syn. n.**
Voria **Other species included in *Voria* Robineau-Desvoidy **
aurifrons Townsend, 1892: 67 (*Plagia*). Holotype male (SEMK). Type locality: USA, Pennsylvania.
aurescens Townsend, 1931: 475 (*Itavoria*). Holotype male (USNM). Type locality: Brazil, São Paulo, Itaquaquecetuba. **Comb. n.**
bakeri Townsend, 1928: 395 (*Hystricovoria*). Holotype male (USNM). Type locality: Philippines, Luzon, Mt. Makiling [as “Mount Maquiling”].** Comb. n.**
munroi Curran, 1938: 6 (*Afrovoria*). Holotype male (SANC). Type locality: South Africa, Mpumalanga, Barberton.
indica Mesnil, 1953: 170 (*Voria *(*Anavoria*)). Holotype female (NHMUK). Type locality: India, Uttarakhand, Dehra Dun.
capensis Villeneuve, 1935 (*Voria*): 138. Holotype male (not located, possibly lost or destroyed). Type locality: South Africa.
setosa Brauer & Bergenstamm, 1891: 409, 439 [also 1891: 105, 135] (*Plagia*) as “*setosa* Wd. litt. Cap. [Cape of Good Hope]”, *nomen nudum*.
micronychia Chao & Zhou, 1993: 1335 (*Voria*). Holotype male (IZCAS). Type locality: China, Yunnan, Zhongdian, 2400m.
operosa Robineau-Desvoidy, 1863: 827 (*Voria*), *nomen dubium.*
parva Johnson, 1919: 436 (*Plagia*). Syntypes, 2 females (not located, possibly lost or destroyed). Type locality: Jamaica, “Liguanea Plain, near Kingston”.
rufitorax Pazos, 1914: 1002 (*Plagia*), *nomennudum*.
pollyclari Rocha-e-Silva, Lopes & Della Lucia, 1999: 85 (*Cyrtophloeba*). Holotype male (UFVB). Type locality: BRASIL, Minas Gerais, Viçosa [20°45′S e 40°51′W]. **Comb. n.**
ruralis Fallén, 1810: 265 (*Tachina*). Lectotype male (NHRS), by designation of Crosskey (1973). Type locality: Sweden, Skåne, Äsperöd [as "Esperöd"]. [Townsend (1939) mentions a holotype; however, this cannot be taken as a lectotype fixation because the specimen was not made distinguishable from the remainder of the type series.]
ambigua Fallén, 1810: 275 (*Tachina*). Holotype female (NHRS or MZLU). Type locality: Sweden.
verticalis Meigen, 1824: 299 (*Tachina*). Holotype male (MNHN). Type locality: Europe.
latifrons Robineau-Desvoidy, 1830: 196 (*Voria*). Syntypes, unspecified number and sex (MNHN). Type locality: France, Gentilly.
arcuata Macquart, 1834: 264 (*Tachina*). Holotype male (MNHL). Type locality: France, Lille**.**
Voria *interruptaTachina*
transversa Macquart, 1848: 96 (*Plagia*). Holotype female (ETHZ). Type locality: Switzerland, Zurich.
spinicosta Palm, 1876: 419 (*Tachina*). Holotype female (not located, possibly lost or destroyed). Type locality: Austria, Innsbruck.
americana van der Wulp, 1890: 102 (*Plagia*). Syntypes, males and females (NHMUK). Type localities: Mexico: Veracruz (Orizaba); Guerrero (Venta del Zopilote); Xucumanatlan; Omilteme and Tabasco (Teapa).
mexicana Giglio-Tos, 1893: 5 (*Plagia*). Holotype female (MRSN). Type locality: Mexico.
brasiliana Townsend, 1929: 380 (*Voria*). Syntypes, males and females (USNM). Type locality: Brazil, São Paulo, Itaquaquecetuba.
edentata Baranov, 1932: 83 (*Voria*). Holotype male (SDEI) (see O'Hara et al. 2009). Type locality: Taiwan, P’ingtung Hsien, Changkou [as “Kankau”, near Hengch’un].
ayerzai Blanchard, 1943: 157 (*Plagia*). Syntypes, unspecified number and sex (MLPA). Type locality: not given [listed as Argentina according to Guimaraes (1971)].
ciliata d’Aguilar, 1957: 261 (as ssp. of*Voria ruralis*). Holotype male (USNM). Type locality: China, Sichuan, Suifu.
saginata Walker, 1861: 298 (*Eurigaster*). Holotype female (NHMUK). Type locality: Mexico.
signata . Incorrect subsequent spelling of *saginata* Walker, 1861 (Guimaraes 1971: 93, 320).
setosa Townsend, 1914: 14 (*Xenoplagia*). Holotype female (USNM). Type locality: Peru, Cañada de Saman, Rio Chira, Peru. **Comb. n.**
Voria Voria latifrons Robineau-Desviody, 1830: 195 [= Tachina ruralis Fallén, 1810: 265]: [= Tachina ruralis Fallén, 1810]

#### Diagnosis

*Voria* can be distinguished by the following combination of traits: compound eye bare; vertex, at its narrowest point, approximately 0.6X eye width in dorsal view; frontal vitta widened posteriorly, ranging in color from dark gray to gold; both sexes with well-developed lateral vertical setae; fronto-orbital plate with 2–4 proclinate orbital setae; fronto-orbital plate ranging from bare to haired; frontal setae descending below base of pedicel; parafacial with some fine hairs on upper 1/3, sometimes with a small tuft of hairs at level of vibrissa; parafacial with 1–3 strong proclinate setae below lowest frontal seta; occiput without black setae posterior to postocular row; genal dilation very slightly developed; prosternum bare; three postsutural supra-alar setae, the anteriormost reduced and much weaker than first postsutural dorsocentral seta; scutellum with four pairs of marginal setae and one pair of erect to semi-erect apical setae; one or two pairs of sub-erect discal setae on scutellum, in line with subapical setae; anepimeral setae not or weakly differentiated from other hairs on anepimeron; wing cell r_4+5_ open at wing margin; bend of vein M with a long stub; wing vein R_1 _setulose along its entire length; R_4+5_ with setulae extending from the fork to just beyond crossvein R-M; abdomen narrow, oval, black in ground color; tergites 3–5 without median discal setae.

#### Distribution

*Voria* is a widespread cosmopolitan genus originally described from Sweden. The genus ranges across the Holarctic, Afrotropical, Australasian, Oriental and Neotropical regions.

#### Ecology

*Voria* has been known to parasitize lepidopteran larvae primarily in the family Noctuidae (and possibly also Pyralidae) (Arnaud 1978). One record from ACG suggests that *Voria* *erasmocoronadoi* **sp. n.** may also parasitize Uraniidae.

### Voria erasmocoronadoi

Fleming & Wood sp. n.

urn:lsid:zoobank.org:act:08890077-723B-4C2A-B7C5-1A9709DA4ADE

#### Materials

- **Type status:** Holotype. **Occurrence:** occurrenceDetails: http://janzen.sas.upenn.edu; catalogNumber: DHJPAR0059183; recordedBy: D.H. Janzen, W. Hallwachs & Keiner Aragon; sex: male; lifeStage: adult; otherCatalogNumbers: ACGBA5600-16, 16-SRNP-45350, BOLD:AAG9377; **Taxon:** scientificName: *Voria* *erasmocoronadoi*; phylum: Arthropoda; class: Insecta; order: Diptera; family: Tachinidae; genus: Voria; specificEpithet: erasmocoronadoi; scientificNameAuthorship: Fleming & Wood, 2017; **Location:** continent: Central America; country: Costa Rica; stateProvince: Guanacaste; county: Sector Rincon Rain Forest; locality: Area de Conservacion Guanacaste; verbatimLocality: Casa Keyner; verbatimElevation: 121; verbatimLatitude: 10.95644; verbatimLongitude: -85.2661; verbatimCoordinateSystem: Decimal; decimalLatitude: 10.95644; decimalLongitude: -85.2661; **Identification:** identifiedBy: AJ Fleming; dateIdentified: 2017; **Event:** samplingProtocol: Reared from the larva of the Noctuidae, *Ctenoplusia* *oxygramma*; verbatimEventDate: 06-Mar-2016; **Record Level:** language: en; institutionCode: CNC; collectionCode: Insects; basisOfRecord: Pinned Specimen
- **Type status:** Paratype. **Occurrence:** occurrenceDetails: http://janzen.sas.upenn.edu; catalogNumber: DHJPAR0006953; recordedBy: D.H. Janzen, W. Hallwachs & Gloria Sihezar; individualCount: 1; sex: female; lifeStage: adult; preparations: pinned; otherCatalogNumbers: ASTAV195-06, 06-SRNP-1503, BOLD:AAG9377; **Taxon:** scientificName: Voria erasmocoronadoi; phylum: Arthropoda; class: Insecta; order: Diptera; family: Tachinidae; genus: Voria; specificEpithet: erasmocoronadoi; scientificNameAuthorship: Fleming & Wood, 2017; **Location:** continent: Central America; country: Costa Rica; countryCode: CR; stateProvince: Alajuela; county: Sector San Cristobal; locality: Area de Conservacion Guanacaste; verbatimLocality: Sendero Carmona; verbatimLatitude: 10.8762; verbatimLongitude: -85.3863; verbatimCoordinateSystem: Decimal; decimalLatitude: 10.8762; decimalLongitude: -85.3863; **Identification:** identifiedBy: AJ Fleming; dateIdentified: 2017; **Event:** samplingProtocol: Reared from the larva of the Noctuidae, *Chrysodeixis* *includens*; verbatimEventDate: 02-Mar-2006; **Record Level:** language: en; institutionCode: CNC; collectionCode: Insects; basisOfRecord: Pinned Specimen
- **Type status:** Paratype. **Occurrence:** occurrenceDetails: http://janzen.sas.upenn.edu; catalogNumber: DHJPAR0007086; recordedBy: D.H. Janzen, W. Hallwachs & Petrona Rios; individualID: DHJPAR0007086; sex: male; lifeStage: adult; preparations: pinned; otherCatalogNumbers: ASTAV328-06, 06-SRNP-30286, BOLD:AAG9377; **Taxon:** scientificName: Voria erasmocoronadoi; phylum: Arthropoda; class: Insecta; order: Diptera; family: Tachinidae; genus: Voria; specificEpithet: erasmocoronadoi; scientificNameAuthorship: Fleming & Wood, 2017; **Location:** continent: Central America; country: Costa Rica; stateProvince: Guanacaste; county: Sector Pitilla; locality: Area de Conservacion Guanacaste; verbatimLocality: Pasmompa; verbatimLatitude: 11.0193; verbatimLongitude: -85.41; verbatimCoordinateSystem: Decimal; decimalLatitude: 11.0193; decimalLongitude: -85.41; **Identification:** identifiedBy: AJ Fleming; dateIdentified: 2017; **Event:** samplingProtocol: Reared from the larva of the Uraniidae, *Erosia* biolep03; verbatimEventDate: 19-Jan-2006; **Record Level:** language: en; institutionCode: CNC; collectionCode: Insects; basisOfRecord: Pinned Specimen
- **Type status:** Paratype. **Occurrence:** occurrenceDetails: http://janzen.sas.upenn.edu; catalogNumber: DHJPAR0057036; recordedBy: D.H. Janzen, W. Hallwachs & Freddy Quesada; individualID: DHJPAR0057036; sex: female; lifeStage: adult; preparations: pinned; otherCatalogNumbers: ACGBA4946-15, 15-SRNP-30148, BOLD:AAG9377; **Taxon:** scientificName: Voria erasmocoronadoi; phylum: Arthropoda; class: Insecta; order: Diptera; family: Tachinidae; genus: Voria; specificEpithet: erasmocoronadoi; scientificNameAuthorship: Fleming & Wood, 2017; **Location:** continent: Central America; country: Costa Rica; stateProvince: Guanacaste; county: Sector Pitilla; locality: Area de Conservacion Guanacaste; verbatimLocality: Sendero Rotulo; verbatimLatitude: 11.0135; verbatimLongitude: -85.4241; verbatimCoordinateSystem: Decimal; decimalLatitude: 11.0135; decimalLongitude: -85.4241; **Identification:** identifiedBy: AJ Fleming; dateIdentified: 2017; **Event:** samplingProtocol: Reared from the larva of the Noctuidae, *Diastema morata*; verbatimEventDate: 08-Feb-2015; **Record Level:** language: en; institutionCode: CNC; collectionCode: Insects; basisOfRecord: Pinned Specimen

#### Description

**Male **(Fig. 2a, b, c). Length: 5–7 mm (n=2). **Head **(Fig. 2b, e): mainly light colored; gena and parafacial with silvery- white pollinosity; frontal vitta gold pollinose; fronto-orbital plate gold pollinose; occiput silvery-white pollinose. Antenna: pedicel dark gray, basally turning to faded orange where it meets the postpedicel; arista dark brown; palpus darkened along basal 2/3, yellow apically. Eye bare; vertex, at its narrowest point, approximately 0.67X as wide as an eye in dorsal view; lateral vertical seta well-developed; ocellar setae proclinate and well-developed, inserted lateral to anterior ocellus; frontal setae descending well into parafacial beyond level of lower margin of pedicel; fronto-orbital plate with three proclinate orbital setae; with short proclinate hairs intermingled among frontal setae and along edges of frontal vitta; three uppermost frontal setae reclinate; parafacial with fine hairs in upper third and a small tuft of hairs slightly above vibrissal angle; one proclinate parafacial seta, below lowermost frontal seta; parafacial at its narrowest point, about as wide as postpedicel; facial ridge straight, with up to three small setae above vibrissa; vibrissa level with facial margin; facial margin not visible in lateral view; gena, in profile, approximately 0.2X height of eye; genal dilation not well developed; occiput slightly convex, with blond setae beyond postocular row; pedicel 0.75X the length of postpedicel; arista bare, basal half twice as thick as apical half. **Thorax **(Fig. 2a, c, d, f): entirely dark gray, legs black; with four, barely visible dorsal vittae on scutum, not visible postsuturally; prosternum bare, proepisternum bare; postpronotum with two strong inner setae plus two shorter and weaker setae, the three basal setae arranged in a line with one weaker seta in front. Chaetotaxy: acrostichal setae 3:3; dorsocentral setae 3:3; intra-alar setae 3:3; three post-sutural supra-alar setae, the anteriormost one very fine and adjacent to suture; second postsutural supra-alar at least twice as wide at base as first postsutural supra-alar (Fig. 2a, d); three katepisternal setae; katepimeron bare; anepimeron with 3–4 setae, weakly differentiated from surrounding hairs (Fig. 2c, f); anatergite setose; scutellum with three pairs of strong marginal setae (basal, lateral and subapical), and one pair of erect apical setae slightly retracted from apex of scutellum (Fig. 2a, d); basal seta 0.75–0.82X as long as lateral seta; subapical setae crossed and horizontal, slightly longer than lateral setae; scutellum with 2–3 rows of erect discal setae in front of apical setae. Anterior and posterior lappets of posterior spiracle usually subequal in size. Wing****(Fig. 2a, d):****membrane hyaline, very slightly infuscate; costal spine absent; vein R_1_ dorsally setulose; vein R_4+5_ dorsally setulose from fork to beyond intersection with crossvein R-M, halfway to margin; vein CuA_1_ bare; bend of vein M with a stub about 0.5X as long as crossvein DM-Cu; crossvein DM-Cu very oblique and slightly sinusoidal; cell r_4+5_ open at wing margin. Legs: black in ground color; medial anterior surface of fore coxa covered with appressed setae; preapical anterodorsal seta on fore tibia much longer than preapical dorsal seta; row of irregularly sized setae present on anterodorsal surface of fore tibia; mid tibia with at least three strong anterodorsal setae; hind tibia with 2–3 dorsal preapical setae; anterodorsal setae on hind tibia irregular in length and thickness. **Abdomen **(Fig. 2a, c, d, f):****black in ground color; T3 and T4 with uniform gray pollinosity extending to tergal edge (when viewed under single point light source this can appear to terminate directly anterior to insertion of marginal setae); T5 with gray pollinosity over 50% of tergum. Abdomen elongate, ovoid, with mid-dorsal depression of ST1+2 extending posteriorly to hind margin of syntergite; ST1+2 without median marginal setae, laterally with a small tuft of slightly thickened setae; T3 with two median marginal setae, lacking discal setae; T4 and T5 each with a row of 6–7 marginal setae; T4 lacking discal setae, T5 with a complete row of discal setae. **Terminalia **(Fig. 3):****posterior margin of sternite 5 with a deeply excavated U-shaped median cleft; lateral lobes of sternite apically squared, with a marginal row of 6–7 setae (Fig. 3d); basal section shorter than apical lobes. Cerci, in posterior view, medially separated and strongly divergent, with a few short setae on basal half (Fig. 3c). In lateral view, cercus sickle-shaped and strongly tapered apically (Fig. 3a), with a medial fork apparent when viewed at an oblique angle (Fig. 1d). Surstylus well-developed, stout basally in lateral view, like a broadly rounded triangle terminating in a small knob, appearing hooked or slightly beaked apically (Fig. 3b); in posterior view, surstyli basally enlarged and apically straight (Fig. 3c). Pregonite plate-like, medially fused; postgonite well-developed, elongate and slender, strongly laterally directed and medially curved so as to appear inwardly hooked when viewed dorsally; postgonite scythe-like, with a slight downward turn in lateral view (Fig. 3b, c). Basiphallus appearing as continuous with epiphallus; basiphallus+epiphallus 0.10X as long as distiphallus; distiphallus long and ribbon-like, apically inflated (Fig. 3a), with a slender, longitudinal, sclerotized parallel reinforcement on each side (Fig. 3a, b), not reaching apex. 

**Female** (Fig. 2d, e, f). length: 5–6 mm (n=2). Identical to the male, differing only in the terminalia.

#### Diagnosis

*Voriaerasmocoronadoi***sp. n.** can be differentiated from its congeners by the following combination of the traits: vertex, at its narrowest point, approximately 0.67X eye width in dorsal view; frontal vitta widened posteriorly, with strong gold pollinosity; fronto-orbital plate gold pollinose, with three proclinate orbital setae; frontal setae descending below base of pedicel, with sparse black hairs intermingled among setae; fronto-orbital hairs extending into upper third of parafacial; and parafacial with only one proclinate seta, below lowest frontal seta. *Voria ruralis* can be differentiated from *V. erasmocoronadoi*by the lack of gold pollinosity on the fronto-orbital plate, and the regular length and spacing of the setae on the anterodorsal surface of the fore tibia. In addition to the barcode and the external morphological character differences, our results also suggest that there are strong differences in the shapes of the postgonite and surstylus between the various populations of *V. ruralis*previously described and our new species. We consider a detailed comparison to exceed the scope of the present paper. *Voria aurifrons* can be distinguished by the presence of a slight infuscation along the costal margin of the wing, and by having legs of a reddish ground color, two character states lacking in *V. erasmocoronadoi*.

#### Etymology

*Voria erasmocoronadoi***sp. n. **is named in honor of Mr. Erasmo Coronado Caballo of Liberia, Costa Rica, in recognition of his years of dedicated logistic support to the Guanacaste Dry Forest Conservation Fund and to the ACG parataxonomist program, participants of which found and reared the caterpillar hosts of this fly.

#### Distribution

Costa Rica, ACG (Guanacaste and Alajuela provinces), 121–1150m.

#### Ecology

*Voria erasmocoronadoi* **sp. n.** has been reared seven times at ACG: six times from caterpillars of three species of Noctuidae, *Ctenoplusia oxygramma *(Geyer, 1832), *Chrysodeixis includens *(Walker, 1858) and *Diastema morata*Schaus, 1894, and once from a caterpillar of *Erosia* *biolep*DHJ03 in the family Uraniidae. Sites of collection include cloud forest, rainforest, and dry-rain intergrade forest.

## Analysis

The DNA barcode region sequences for *Voria* are AT-biased (70%), as expected for insect DNA. The sequences displayed no heteroplasmy or stop codons that would suggest the amplification of a pseudogene and we conclude that these barcodes are mtDNA for *Voria*. There are currently five other species in BOLD provisionally identified as *Voria* *ruralis* from localities other than ACG in BOLD, each with their own unique Barcode Index Numbers (BINs); all are likely to be members of the *Voria ruralis*species complex. The sequence variation between *V. erasmocoronadoi***sp. n.****(BOLD:AAG9377) and the other *Voria *BINS on BOLD ranges from 3 to 11%. Based on our experience with other species complexes of morphologically indistinct insect species groups in ACG, this distance is highly supportive of the status of *V. erasmocoronadoi***sp. n. **as a separate species*. *

## Supplementary Material

XML Treatment for Voria

XML Treatment for Voria erasmocoronadoi

## Figures and Tables

**Figure 1a.**
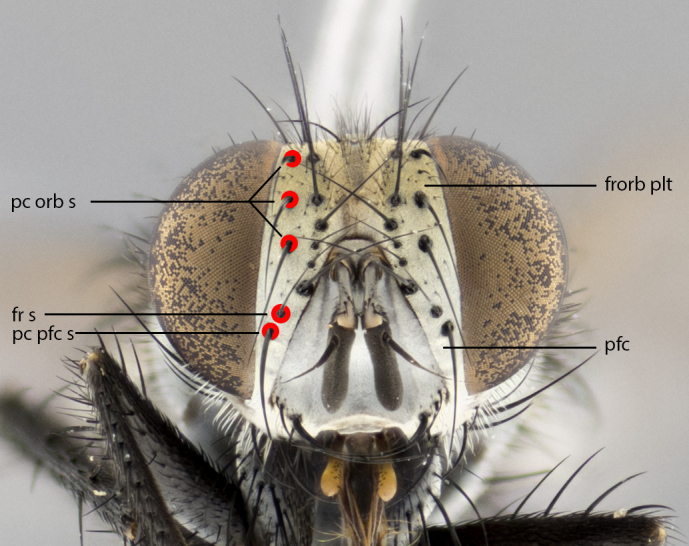
frontal view of head, showing landmark features. Abbreviations: frorb plt: fronto-orbital plate; fr s: frontal seta (lowermost highlighted); pc pfc s: proclinate parafacial seta; pfc: parafacial; pc orb s: proclinate orbital seta.

**Figure 1b.**
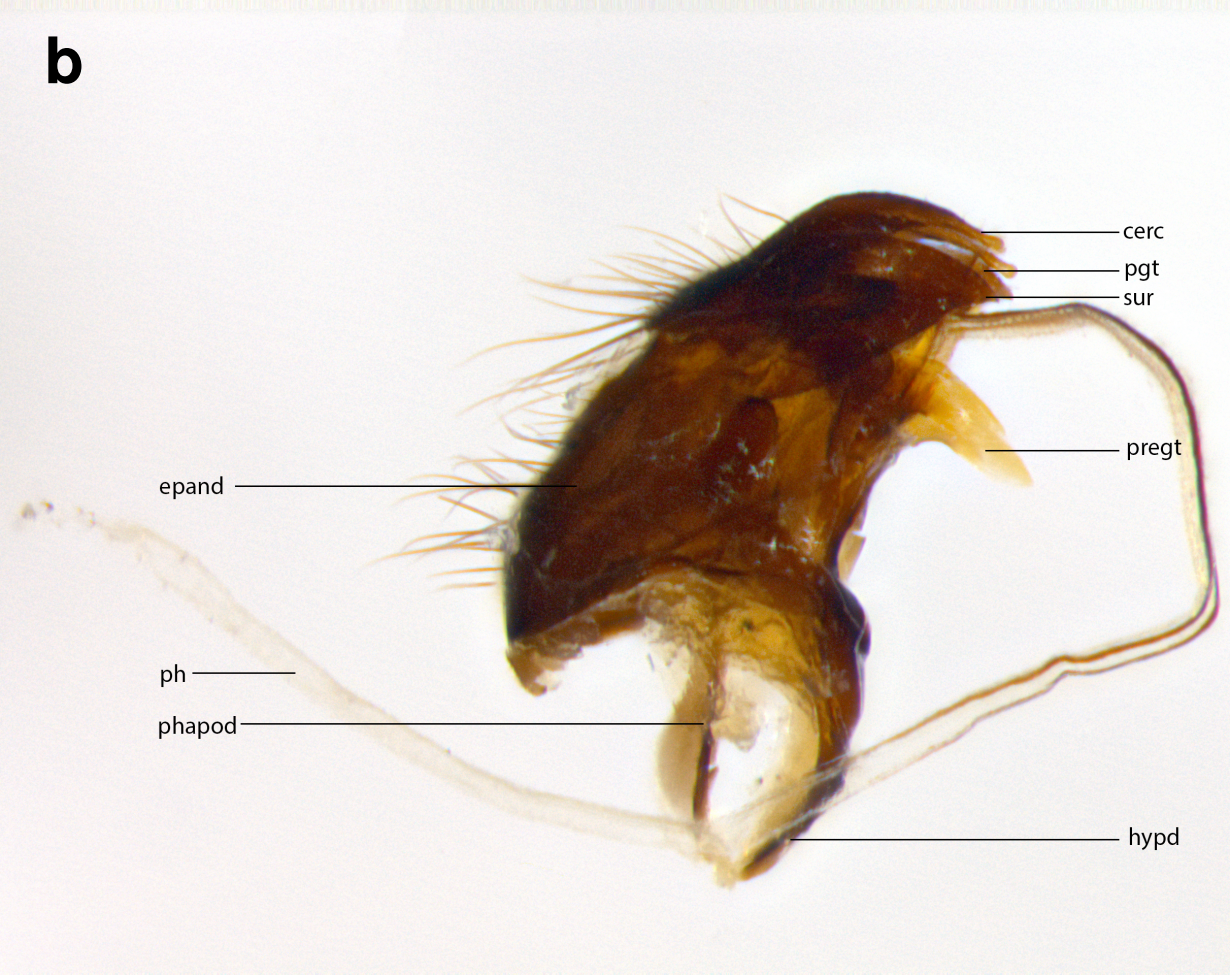
terminalia in lateral view. Abbreviations: cerc: cercus; epand: epandrium; hypd: hypandrium; ph: phallus; phapod: pallapodeme; pgt: postgonite; pregt: pregonite; sur: surstylus

**Figure 1c.**
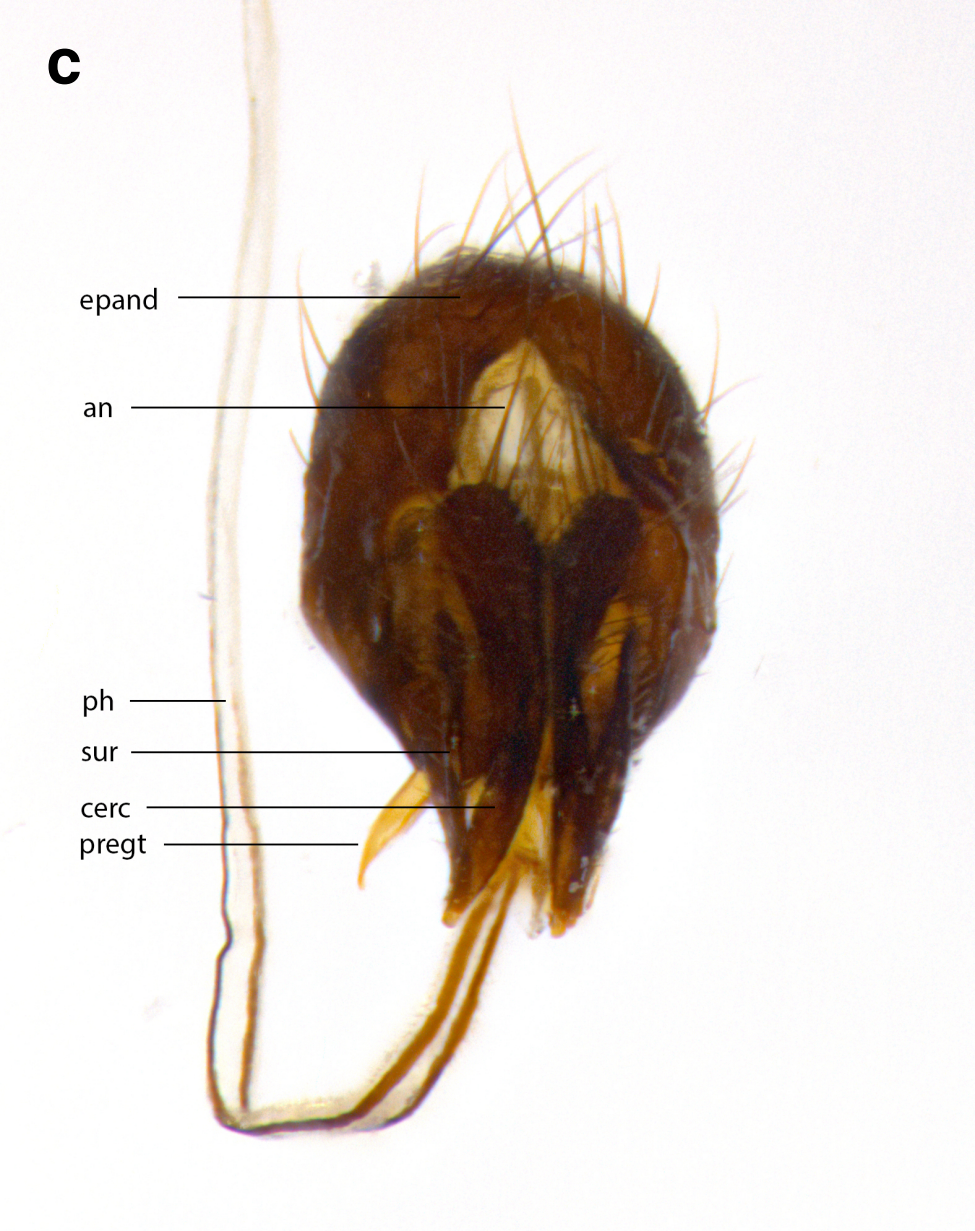
terminalia in dorsal view. Abbreviations: an: anus; cerc: cercus; epand: epandrium; ph: phallus; pregt: pregonite; sur: surstylus

**Figure 1d.**
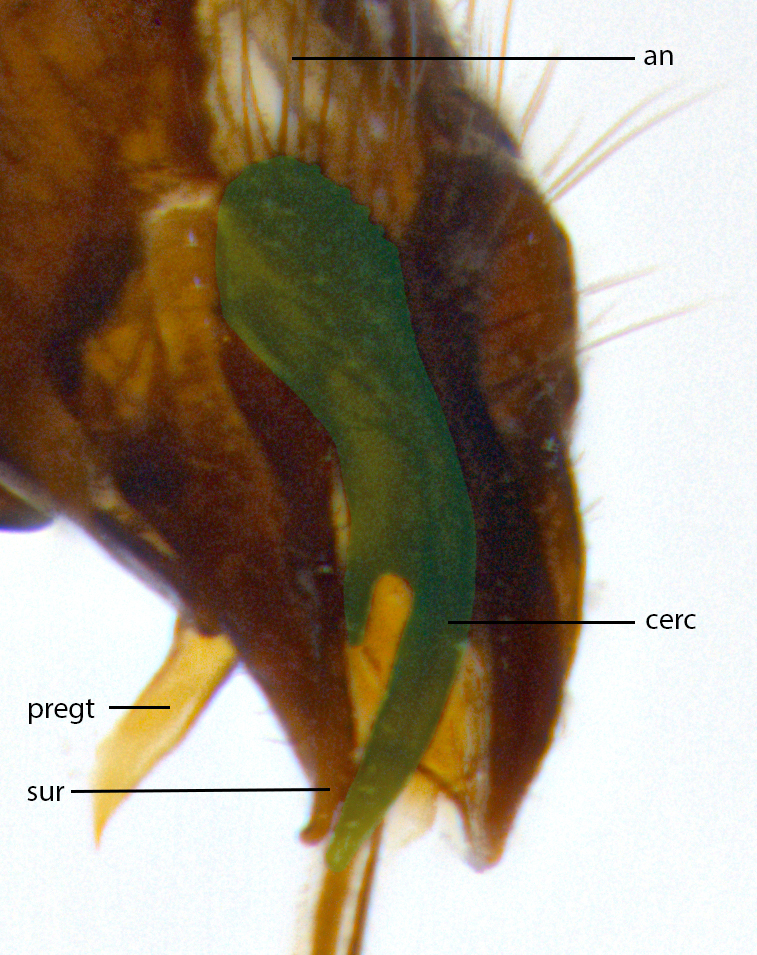
terminalia in oblique view. Abbreviations: an: anus; cerc: cercus; pregt: pregonite; sur: surstylus. Green overlay highlighting forked aspect of cercus.

**Figure 2a.**
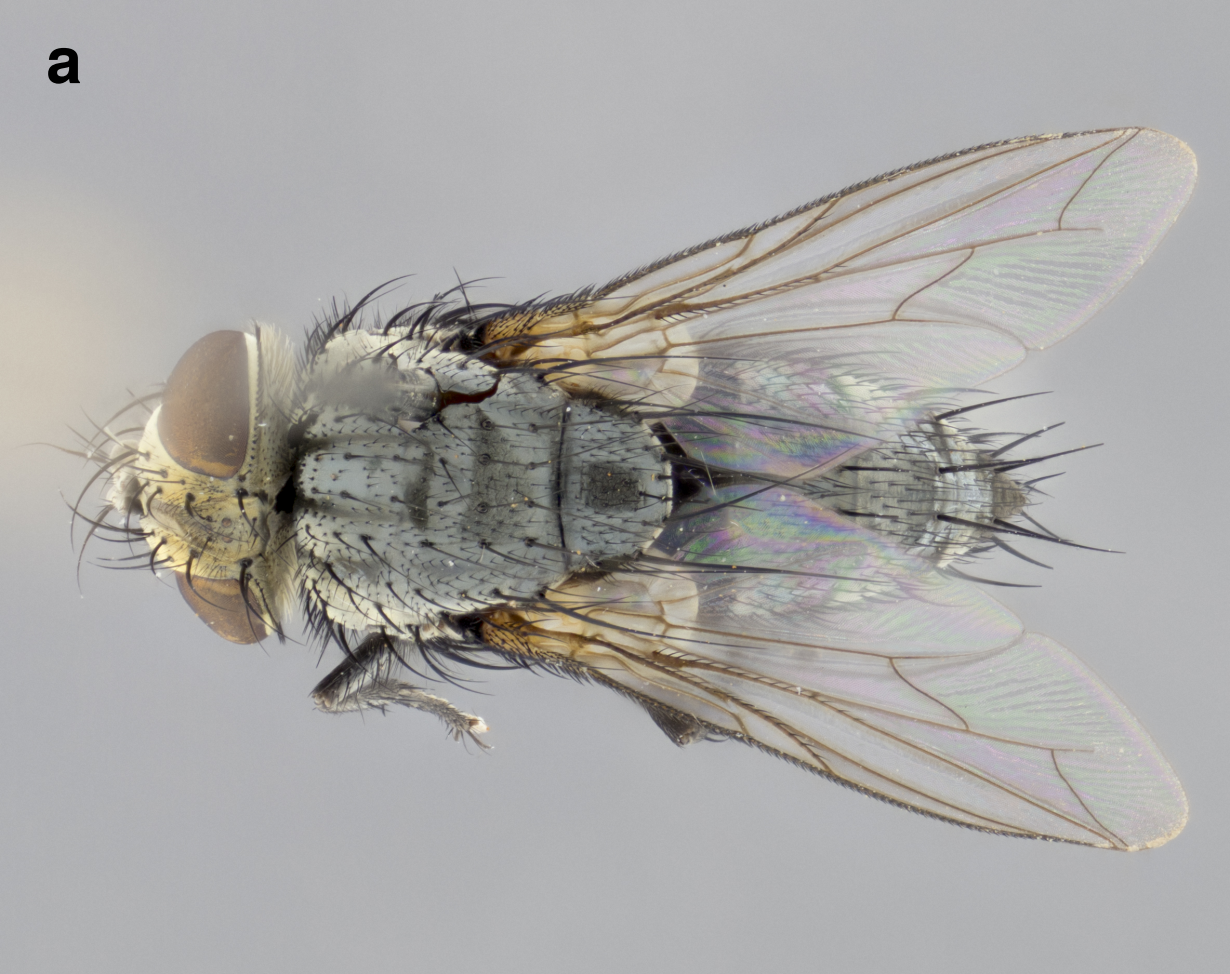
habitus in dorsal view

**Figure 2b.**
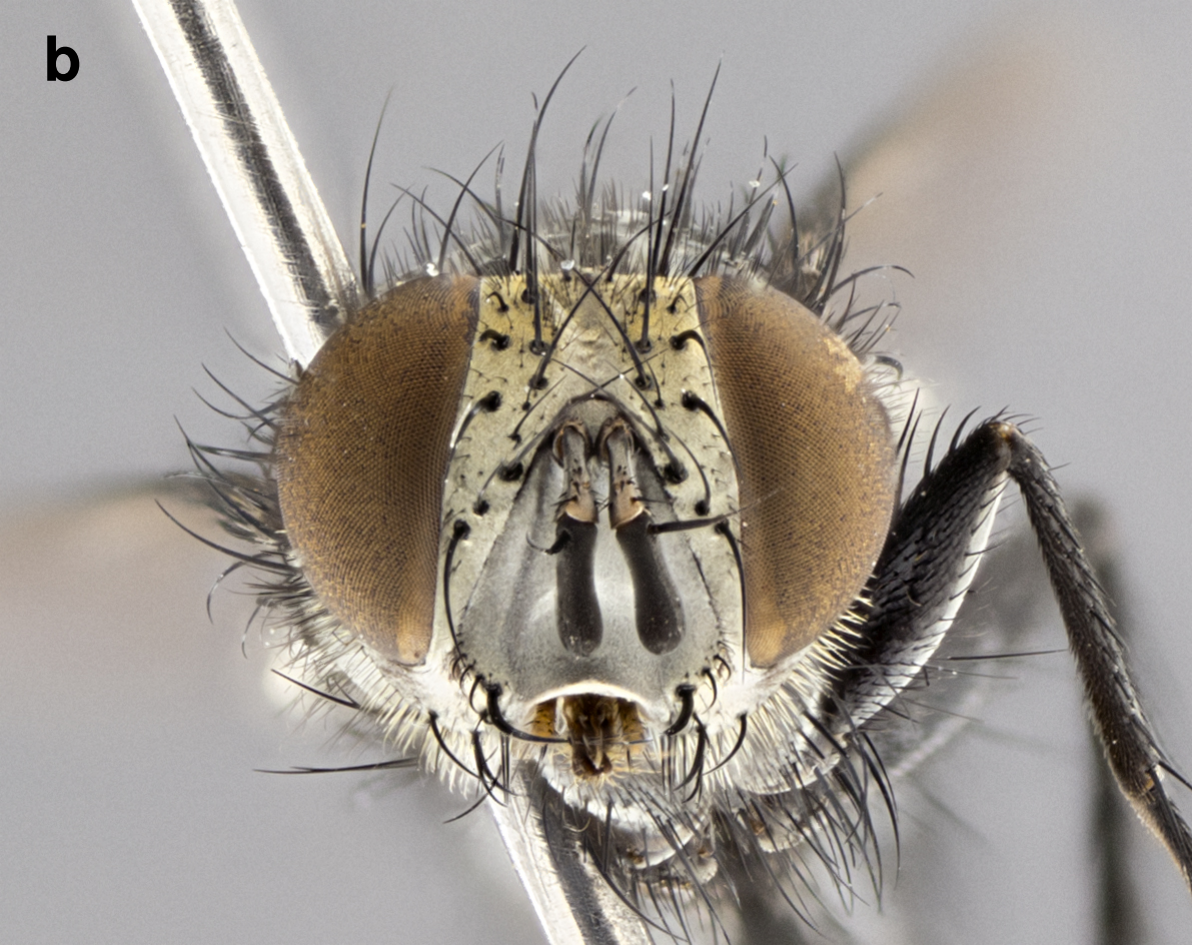
head in frontal view

**Figure 2c.**
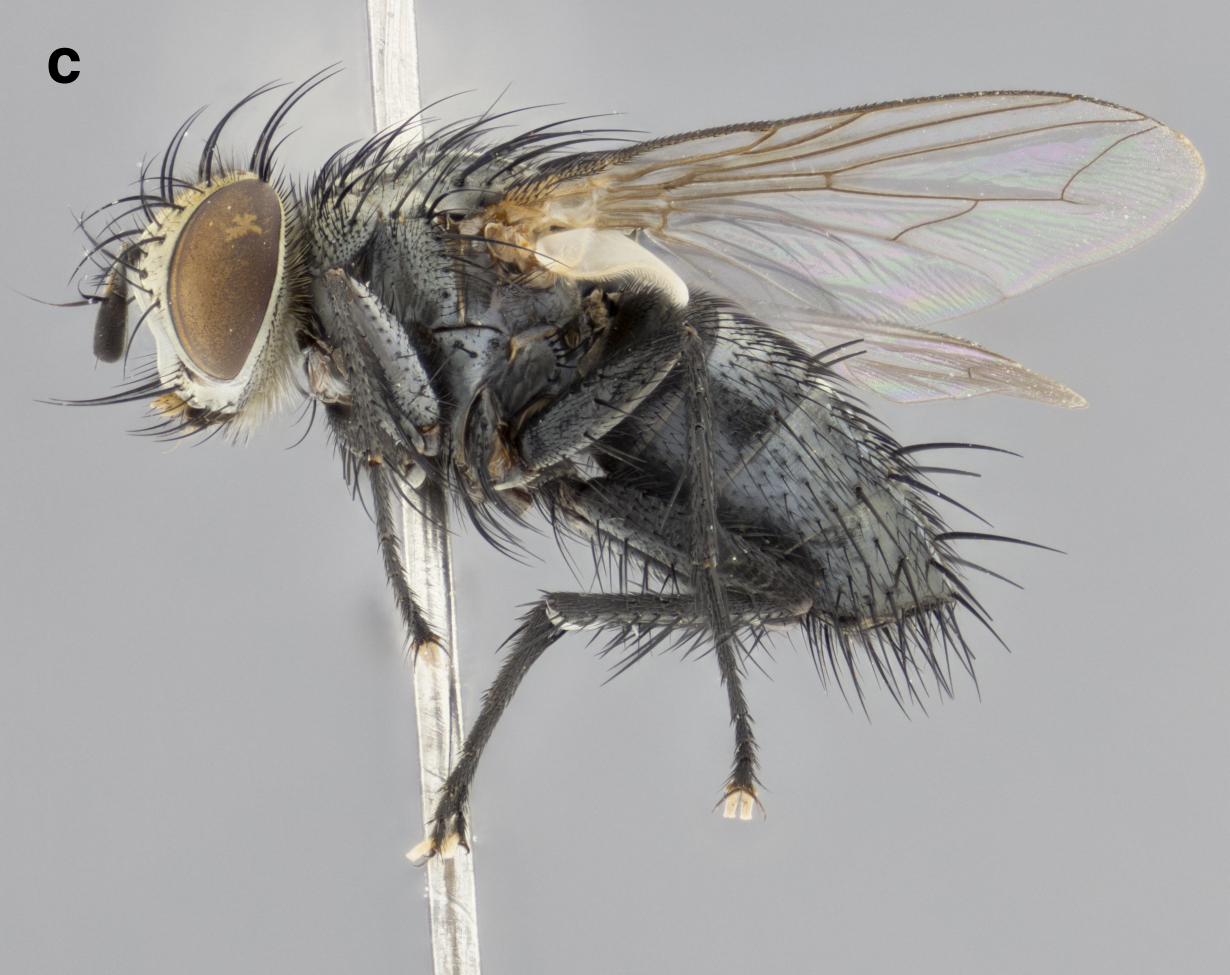
habitus in left lateral view

**Figure 2d.**
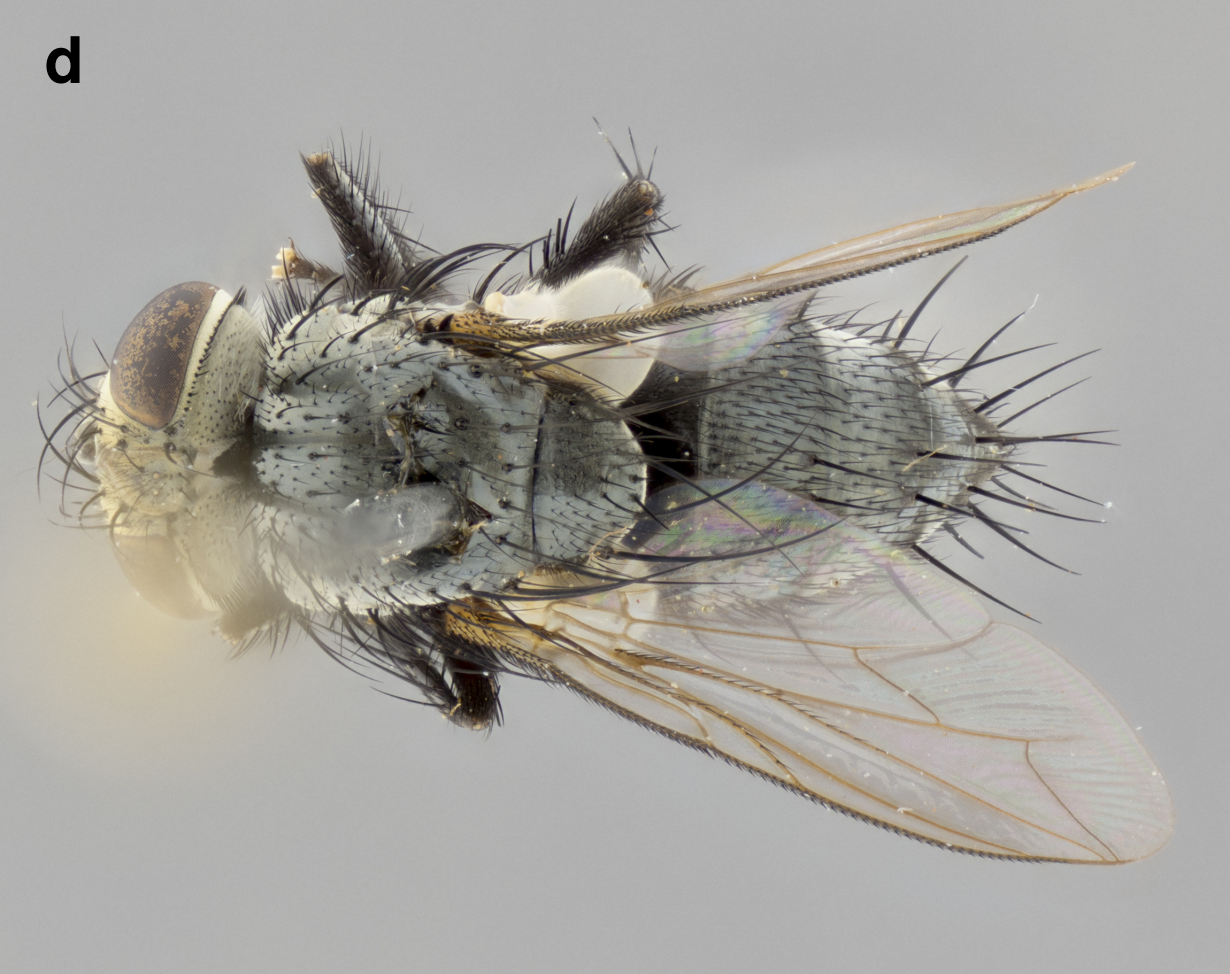
habitus in dorsal view

**Figure 2e.**
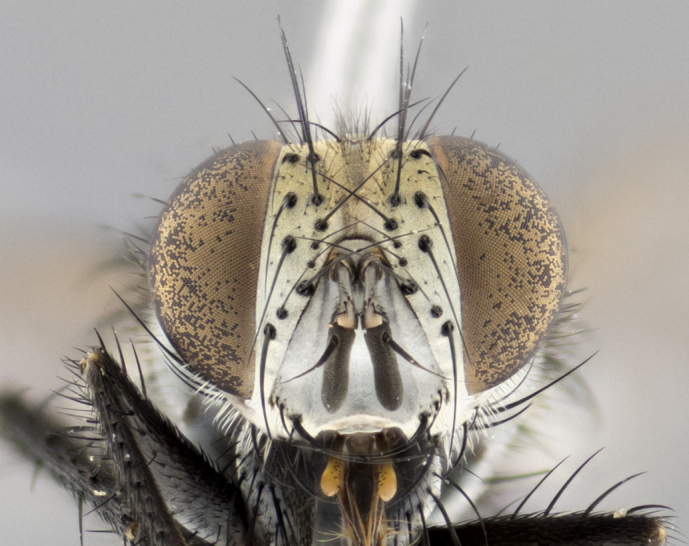
head in frontal view

**Figure 2f.**
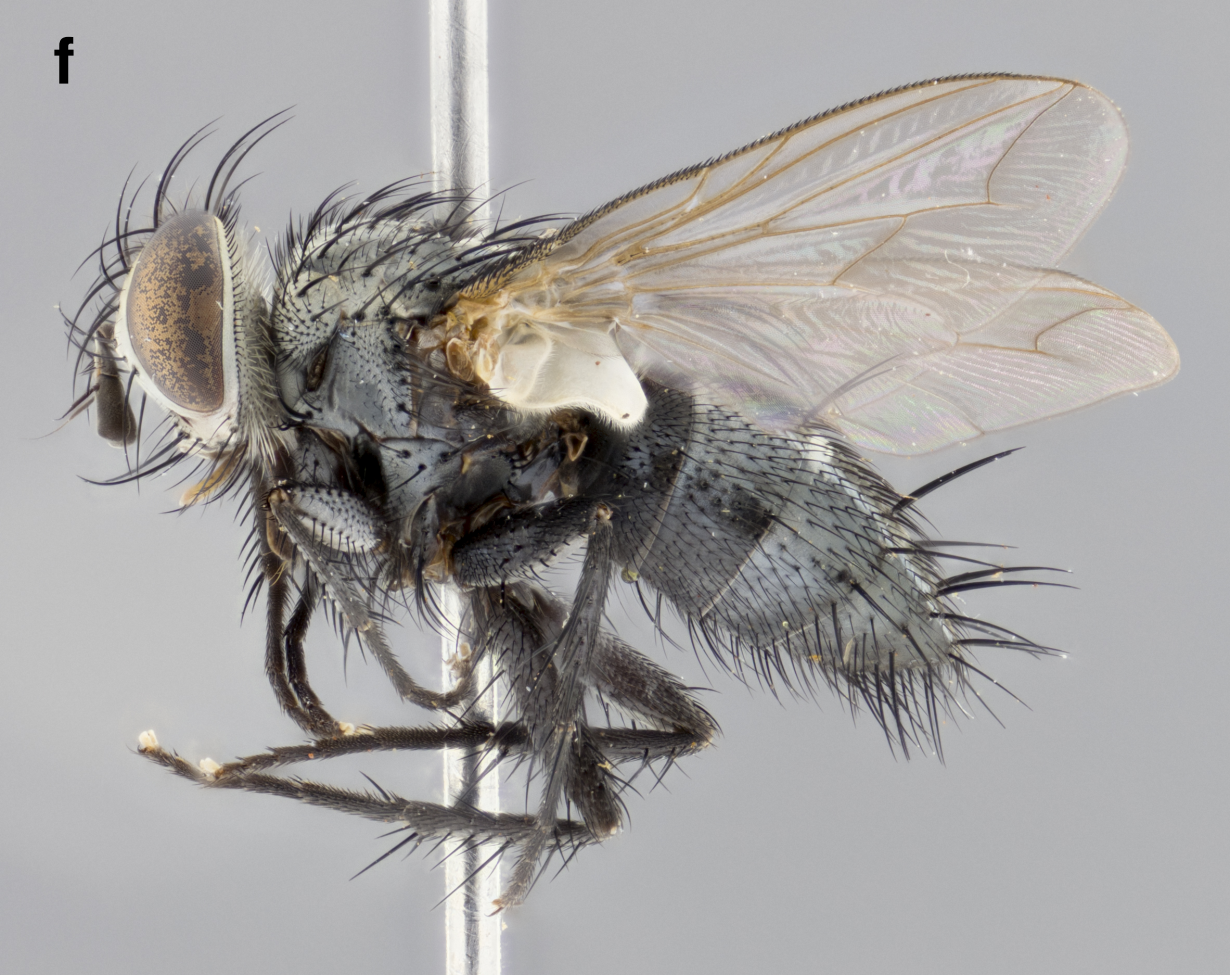
habitus in left lateral view

**Figure 3a.**
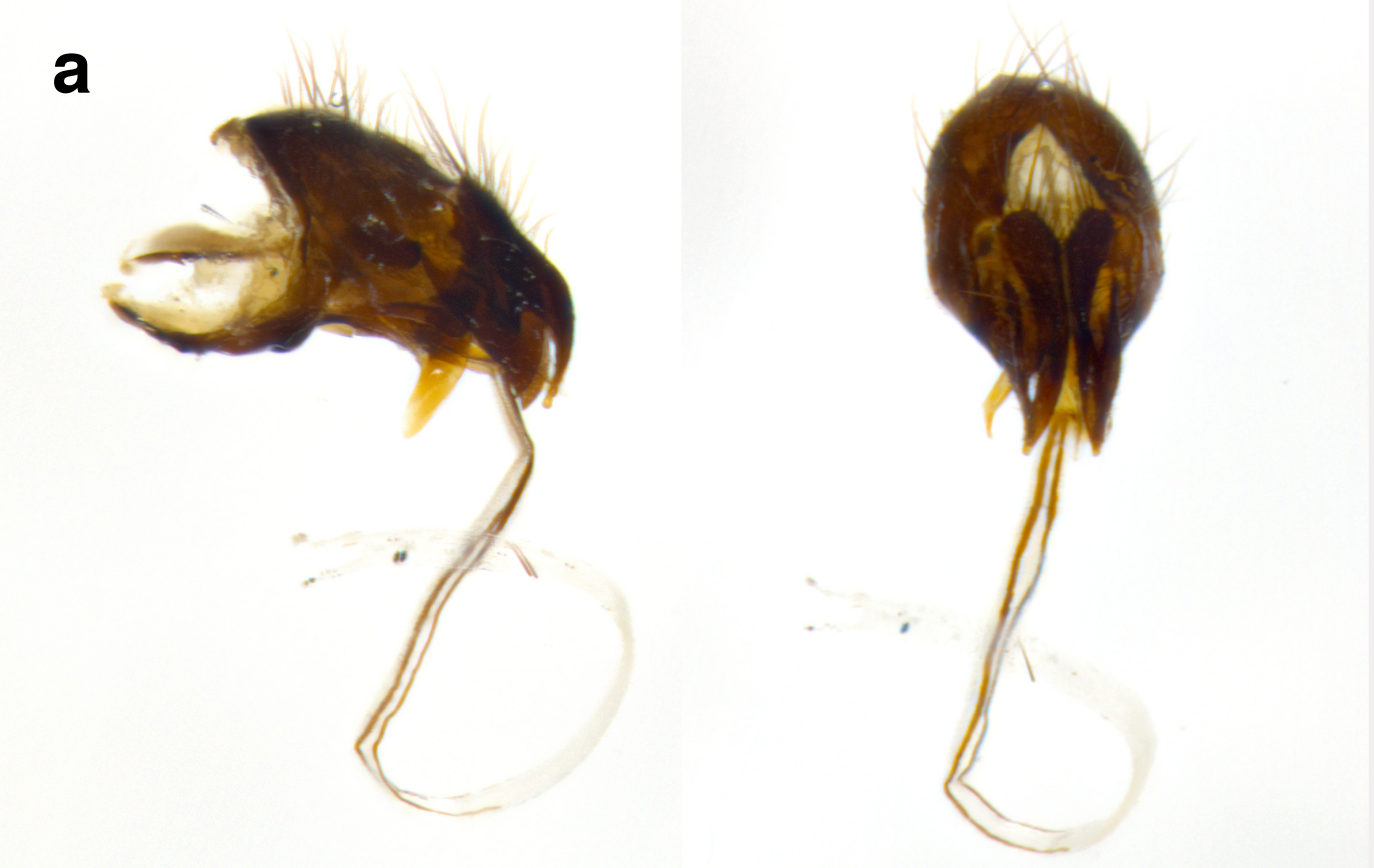
terminalia in lateral and posterior views, showing the relative length of the ribbon-like distiphallus

**Figure 3b.**
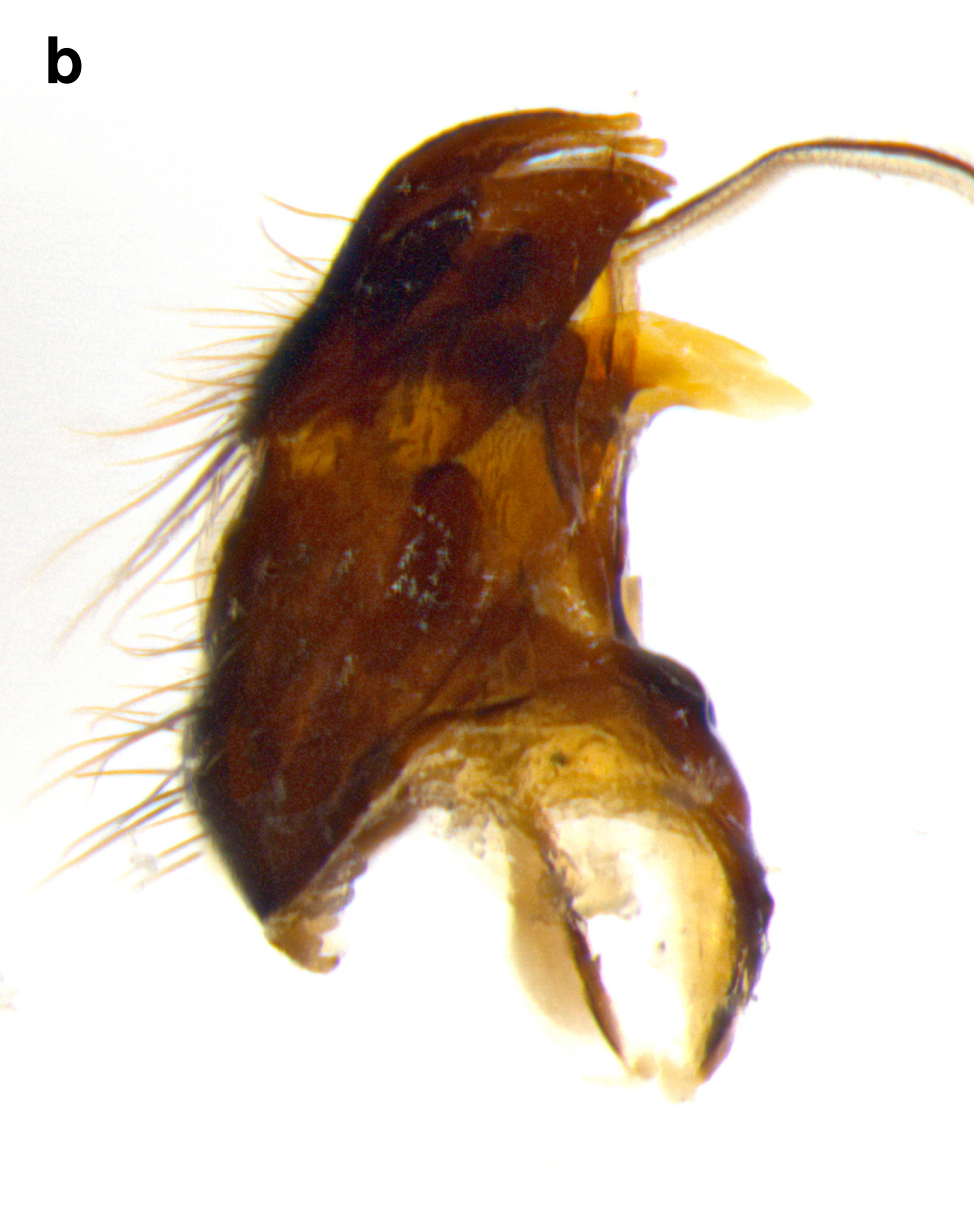
terminalia in lateral view

**Figure 3c.**
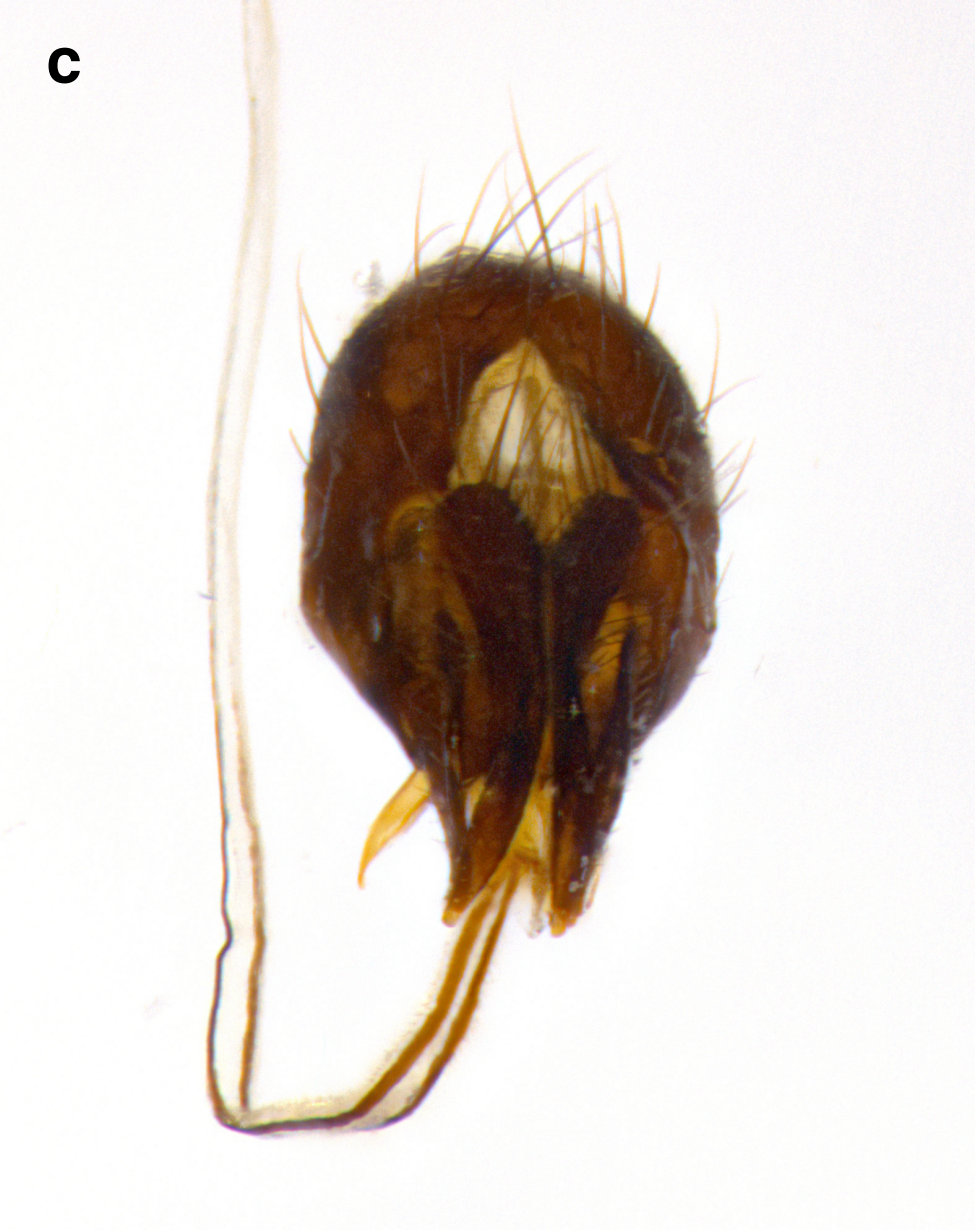
terminalia in posterior view

**Figure 3d.**
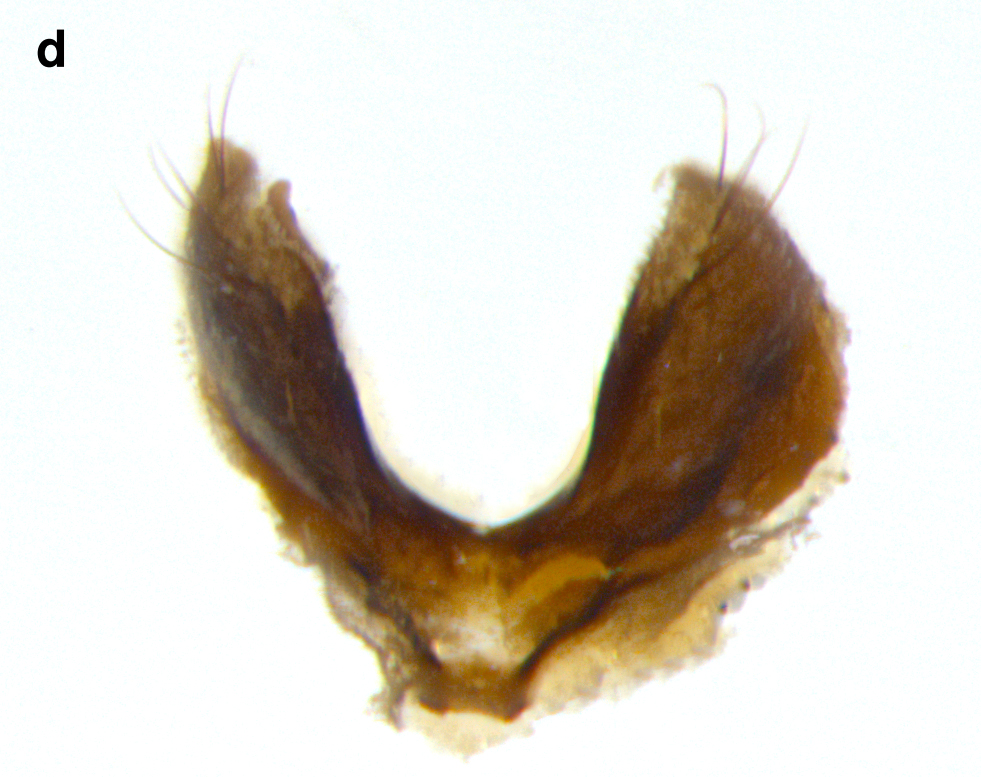
sternite 5 in ventral view
